# RNA polymerase II trapped on a molecular treadmill: Structural basis of persistent transcriptional arrest by a minor groove DNA binder

**DOI:** 10.1073/pnas.2114065119

**Published:** 2022-01-12

**Authors:** Juntaek Oh, Tiezheng Jia, Jun Xu, Jenny Chong, Peter B. Dervan, Dong Wang

**Affiliations:** ^a^Division of Pharmaceutical Sciences, Skaggs School of Pharmacy and Pharmaceutical Sciences, University of California San Diego, La Jolla, CA 92093;; ^b^Division of Chemistry and Chemical Engineering, California Institute of Technology, Pasadena, CA 91125;; ^c^Department of Cellular and Molecular Medicine, University of California San Diego, La Jolla, CA 92093;; ^d^Department of Chemistry and Biochemistry, University of California San Diego, La Jolla, CA 92093

**Keywords:** RNA polymerase II, transcription blockage, minor groove, pyrrole-imidazole polyamide, transcription factor

## Abstract

Elongating RNA polymerase II (Pol II) can be paused or arrested by a variety of obstacles. These obstacles include DNA lesions, DNA-binding proteins, and small molecules. Hairpin pyrrole-imidazole (Py-Im) polyamides bind to the minor groove of DNA in a sequence-specific manner and induce strong transcriptional arrest. Remarkably, this Py-Im–induced Pol II transcriptional arrest is persistent and cannot be rescued by transcription factor TFIIS. In contrast, TFIIS can effectively rescue the transcriptional arrest induced by a nucleosome barrier. The structural basis of Py-Im–induced transcriptional arrest and why TFIIS cannot rescue this arrest remain elusive. Here we determined the X-ray crystal structures of four distinct Pol II elongation complexes (Pol II ECs) in complex with hairpin Py-Im polyamides as well as of the hairpin Py-Im polyamides–dsDNA complex. We observed that the Py-Im oligomer directly interacts with RNA Pol II residues, introduces compression of the downstream DNA duplex, prevents Pol II forward translocation, and induces Pol II backtracking. These results, together with biochemical studies, provide structural insight into the molecular mechanism by which Py-Im blocks transcription. Our structural study reveals why TFIIS fails to promote Pol II bypass of Py-Im–induced transcriptional arrest.

## Significance

Hairpin pyrrole-imidazole (Py-Im) polyamides can be programmed to bind a broad repertoire of DNA sequences. Py-Im small molecules can be used to target cancer-specific coding regions and block transcription elongation. This transcription blockage by Py-Im cannot be rescued by transcription elongation factors, such as TFIIS. The mechanism by which Py-Im blocks transcription remains elusive. To understand the structural basis of this strong transcription blockage, we solved five different structures containing an eight-ring hairpin Py-Im bound with either a Pol II elongation complex (EC) or a DNA duplex. These structures revealed that Py-Im can trap Pol II EC in a treadmill-like manner. This knowledge may pave the way for the development of small molecules that inhibit transcriptional addiction in cancer.

Eukaryotic RNA polymerase II (Pol II) transcription elongation process is subject to pausing and arrest by various regulatory factors and obstacles, such as pause-inducing DNA sequences or secondary structures, DNA modifications, DNA lesions, DNA-binding proteins, or DNA-binding small molecules (1–14). The fate of Pol II is determined by the nature of obstacles and whether this Pol II pausing/arrest can be resolved by other transcription factors in a timely manner. For example, Pol II can bypass natural DNA-pausing sequences, small DNA lesions or modifications, and nucleosome barriers, with the help of other transcription factors, such as Spt4/5, TFIIS, or CSB (5, 7, 15–21). In contrast, Pol II gets arrested by helix distorting bulky DNA lesions (8, 16, 22–28) and certain types of DNA-binding small molecules, such as pyrrole-imidazole polyamides (Py-Im) (18, 29). Intriguingly, transcription factors TFIIS and Spt4/5 cannot rescue the prolonged Pol II arrest induced by Py-Im (18, 29).

The binding of Py-Im to its target DNA sequences has several features. The sequence selectivity of Py-Im is governed by side-by-side arrangement of *N*-methylpyrrole (Py) and *N*-methylimidazole (Im) and the corresponding functional groups on the DNA minor groove floor (30–32). For example, Im/Py recognizes the G/C base pair while Py/Py recognizes A/T or T/A base pairs (30). Hairpin Py-Im oligomers bind to target DNA sequences with high affinity (at nanomolar range) comparable to transcription factors and nucleosomes (30). Hairpin Py-Im oligomers function as a molecular wedge causing the expansion of the minor groove and compression of the major groove of DNA (33, 34).

These features of Py-Im oligomers led to several studies on the inhibition of specific gene expression in colon, cervical, and prostate cancer (35–40). For example, the Py-Im polyamide ARE-1 targets androgen receptor (AR) consensus element and directly competes with AR to decrease the occupancy of AR in the KLK3 promoter and enhancer (37). As a result, ARE-1 can greatly reduce the expression level of KLK3 in enzalutamide-resistant LNCaP cells (37). Similarly, Py-Im **1** targets estrogen response elements and reduces the expression of estrogen receptor–controlled luciferase in breast cancer tumor xenografts (36). In addition to targeting the promoter and enhancer regions, our previous results showed that Py-Im induces strong transcriptional arrest of the elongation complex (EC) in a sequence-specific manner, paving the way to target transcription of specific genes (16, 18, 29). Indeed, Py-Im can also be used to target cancer-specific coding region, such as the gene body of cancer-driving mutant E545K in PIK3CA, a hotspot mutation that occurs in 23 to 36% of cervical cancer cases (41). The alkylating polyamide P3AE5K binds to the coding region of E545K mutation and specifically reduces the expression level of PIK3CA E545K mRNA and protein, leading to apoptotic cell death (38).

The structural mechanism of Py-Im–induced persistent transcriptional arrest remains elusive. In this study, we solved seven X-ray crystal structures, including a double-stranded DNA complexed with hairpin Py-Im **1** and four different Pol II elongation complexes bound with Py-Im **1**. Our structural and functional analyses showed that hairpin Py-Im **1** traps Pol II elongation complex in the n-5 to n-3 position, which forces the elongation complex to be trapped on a futile “treadmill” where Pol II moves back and forth by repetitive extension and cleavage in the presence of TFIIS.

## Results and Discussion

### Py-Im Induces Strong Transcriptional Pausing at Both Full-Bubble Scaffold and Miniscaffold.

**Fig. 1.**
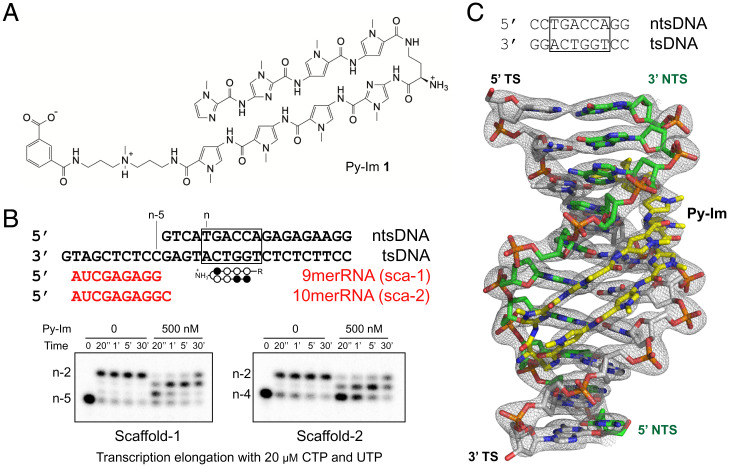
Structural and functional analysis of hairpin Py-Im **1**. (*A*) Chemical structure of hairpin Py-Im **1**. (*B*) Py-Im blocks transcription elongation. Miniscaffolds used for the biochemical assay (*Top*). Transcription elongation assays were performed with scaffolds containing either a 9mer RNA (*Bottom Left*, scaffold-1) or a 10mer RNA (*Bottom Right*, scaffold-2). CTP and UTP were added for transcription elongation. Strong pausing at n-4 and n-3 positions was observed in the presence of Py-Im. (*C*) The crystal structure of Py-Im–DNA duplex complex at 1.8-Å resolution. The 2Fo-Fc electron density map is contoured at 1.3 σ. DNA sequence with the Py-Im–binding site highlighted in the black box (*Top*).

We previously showed that hairpin Py-Im **1** (Fig. 1*A*) induces consecutive Pol II pausing/arrest using the scaffolds containing a fully matched transcription bubble (full-bubble scaffolds) (29). These featuring pausing/arrest bands are located upstream of the actual Py-Im–binding site (i.e., from the n-5 to n-2 positions, where n is the first 5′-base of the hairpin Py-Im–binding site). As the first step toward obtaining the structural insights into Py-Im–induced Pol II arrest, we tested whether we could recapitulate these Py-Im–induced pausing/arrests using the miniscaffold for structural studies. To this end, we designed a miniscaffold harboring a hairpin Py-Im **1** binding sequence (5′-TGACCA-3′) (Fig. 1*B*). This template strand (TS)-binding orientation puts the γ-turn moiety of hairpin Py-Im **1** facing the leading edge of transcribing Pol II (Fig. 1*B*). We performed transcription assays with two different primers (scaffold-1; 9mer RNA, n-5 and scaffold-2; 10mer RNA, n-4) and found that Py-Im **1** induces strong transcriptional pausing/arrest at the miniscaffold in a similar manner as that with the full-bubble scaffolds (Fig. 1*B*) (29). Among all pausing/arrest bands, the n-3 is the strongest pausing/arrest site (Fig. 1*B*).

### Crystal Structure of a Hairpin Py-Im–dsDNA Complex at 1.8-Å Resolution.

The detailed atomic interaction between hairpin Py-Im **1** and DNA duplex is obscured due to a lack of high-resolution crystal structure of hairpin Py-Im **1** bound with dsDNA complex. Here we obtained a high-resolution crystal structure of the hairpin Py-Im **1**–dsDNA (5′-CCTGGTCAGG-3′) complex at 1.8-Å resolution (Fig. 1*C* and *SI Appendix*, Fig. S1*A*). We observed an intensive hydrogen bonding network that stabilizes the hairpin Py-Im **1**–dsDNA complex (*SI Appendix*, Fig. S1*B*). Our structure shows the conserved base recognition pattern between Py-Im and DNA base pairs: Im/Py recognizes G/C and Py/Py recognizes A/T or T/A (*SI Appendix*, Fig. S1*B*) (33, 34, 42, 43). The binding of hairpin Py-Im **1** leads to minor groove widening as well as major groove compression of the DNA duplex in a similar manner as cyclic Py-Im molecules (*SI Appendix*, Fig. S1*C*) (33, 34, 42, 43). This high-resolution structure hairpin Py-Im–dsDNA complex was used as a starting model for building the downstream Py-Im–dsDNA region of the Pol II EC–Py-Im complex and paved the way for our structural studies of Py-Im–induced Pol II–arrested complexes.

### Comparison of Hairpin Py-Im–dsDNA Complex and Pol II–Py-Im Encounter Complex.

To understand the molecular mechanism of Py-Im–induced transcription pausing/arrest in a stepwise manner, we determined six Pol II elongation complex structures. These structures include four distinct Py-Im–bound Pol II elongation complexes and two elongation complexes (*SI Appendix*, Table S1). We observed substantial structural rearrangements of Pol II–Py-Im complexes induced by Py-Im **1**.

**Fig. 2.**
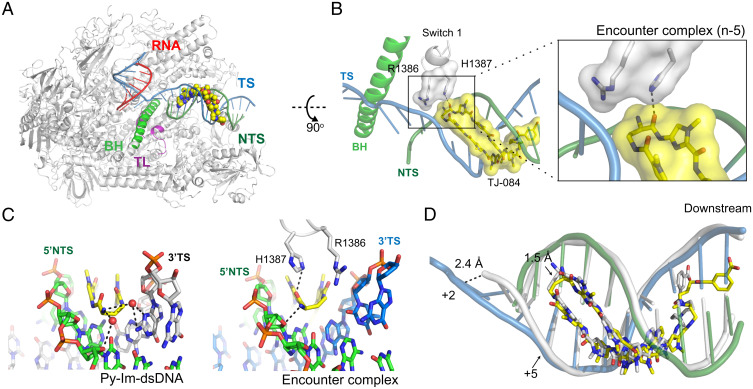
Structural comparison of the Py-Im–dsDNA and Pol II–Py-Im encounter complex. (*A*) Overall structure of the Pol II–Py-Im encounter complex. Pol II is shown in gray (with part of Rpb2 omitted for clarity). RNA (red), template DNA strand (TS, blue), nontemplate DNA strand (NTS, green), bridge helix (BH, green helix), and trigger loop (TL, magenta) are highlighted and labeled. Py-Im molecule is shown as spheres. (*B*) Rotated, enlarged view showing interaction between Py-Im (yellow surface) and the switch 1 motif of Pol II (white surface). Hydrogen bond is shown in black dashed line. (*C*) Hydrogen bond interaction network in the Py-Im–dsDNA (*Left*) and Pol II–Py-Im encounter complex (*Right*). (*Left*) Water-mediated hydrogen bonding between (R)-α-amine-γ-turn tip of Py-Im and DNA minor groove in Py-Im–dsDNA. (*Right*) (R)-α-amine-γ-turn tip of Py-Im is sandwiched with the DNA minor groove and Pol II via direct hydrogen bonding in the Pol II–Py-Im encounter complex. (*D*) Structure superposition of the Py-Im–dsDNA complex and Py-Im encounter complex. Least squares fit superpose was applied using NTS sequence 5′-TGACCA-3′ in COOT (52).

We first solved the Pol II–Py-Im paused complex at the n-5 position (scaffold-1), referred to as the Pol II–Py-Im encounter complex. The encounter complex represents the structural snapshot of when Pol II first senses the approaching Py-Im molecule (Fig. 2 *A* and *B*). By comparing the Pol II–Py-Im encounter complex and the Py-Im–dsDNA complex, we observed substantial changes in terms of molecular interactions and structural rearrangement. First, the molecular interaction network surrounding the γ-turn moiety of hairpin Py-Im **1** is very different. In the Py-Im–dsDNA structure, we found that the hydrogen bonding network interactions between the γ-turn moiety of hairpin Py-Im **1** (α-ammonium tip) and the DNA minor groove are mediated by two ordered water molecules (Fig. 2*C*). By contrast, in the Pol II–Py-Im encounter complex structure, the γ-turn moiety of hairpin Py-Im **1** is sandwiched by two direct hydrogen bonds with DNA and the side chain of Pol II Rpb1 His1387 from the switch region (Fig. 2*C*). This structure confirms our prediction from previous functional and modeling analyses that the Rpb1 switch region is involved in the interaction with the approaching γ-turn of Py-Im (29). Second, we noticed a 2.4-Å shift of the DNA backbone of a nontemplate strand (NTS) nearby the n-5 position in the Pol II–Py-Im encounter complex in comparison with that in the Py-Im–dsDNA complex (Fig. 2*D*). Third, we observed an approximate 1.5- to 1.9-Å shift of the Py-Im molecule toward the downstream DNA in the Pol II–Py-Im encounter complex structure in comparison with that in the Py-Im–dsDNA structure (Fig. 2*D*). It is noteworthy that despite this downstream shift, all key hydrogen bonds are maintained, including those between amide NH and purine N3 and pyrimidine O2 lone pairs, which consist of DNA minor groove “floor” interactions, together with an imidazole lone pair and exocyclic amine of guanine (N2 hydrogen of G) (*SI Appendix*, Figs. S1*B* and S2*A*). These interactions dictate the specificity of Py-Im to its target DNA. Interestingly, in addition to those conserved hydrogen bonds, amide NH adjacent to the imidazole group was in a hydrogen bonding distance with exocyclic amine of guanine (N2 of G) in the Pol II–Py-Im encounter complex.

### Py-Im Induces a 1-bp Downstream DNA Compression in the Pol II–Py-Im Encounter Complex.

**Fig. 3.**
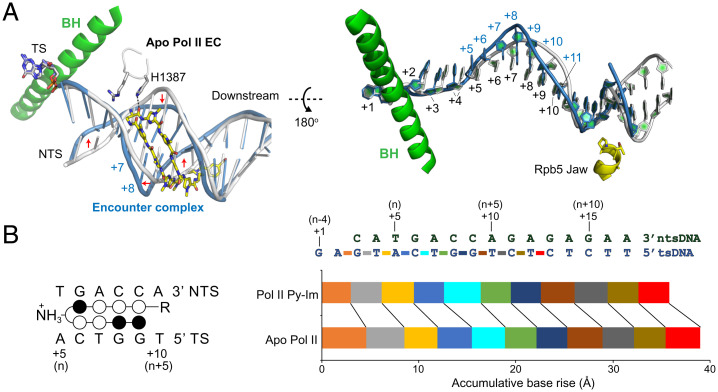
Structural comparison of canonical Pol II elongation complex (EC) and Py-Im encounter complex. (*A*) Structure superposition of the Py-Im encounter complex and canonical Pol II elongation complex (apo, in the absence of Py-Im). The downstream DNA duplex in apo Pol II EC and Pol II–Py-Im encounter complex are shown in white and blue, respectively. Major structural changes in the DNA backbone are highlighted with red arrows. The positions of the downstream TS are labeled. For example, +1 refers to the i+1 template position, the template base in the Pol II active site that is read for pairing with incoming nucleotide substrate. (*B*) Comparison of accumulative base rise parameters of the downstream DNA in the Py-Im encounter complex and canonical Pol II elongation complex (apo). Note that the difference of parameter between apo Pol II EC and Pol II Py-Im was 3.2 Å. (*Left*) The position of the Py-Im–binding site relative to the Pol II active site, where n is the first 5′-base of the hairpin Py-Im–binding site.

To accommodate hairpin Py-Im **1**, we found substantial rearrangement of downstream DNA in both NTSs and TSs of the encounter complex (n-5) (red arrows in Fig. 3*A*). Binding of hairpin Py-Im **1** to Pol II elongation complex induces minor groove widening and major groove narrowing at the downstream DNA (Fig. 3*A* and *SI Appendix*, Fig. S2*B*). In contrast, binding of Py-Im does not change the structure of the Pol II active site and upstream DNA/RNA (Fig. 3*A* and *SI Appendix*, Fig. S2*C*). By superposing the structures of Pol II elongation complexes in the absence (apo Pol II EC) and presence of Py-Im (Pol II–Py-Im encounter complex), we found that there is a 1-bp squeezing in the downstream DNA that occurred upon Py-Im binding (Fig. 3). We found that, upon Py-Im binding, an accumulative base rise from i+2 to i+13 nt of tsDNA was decreased by 3.2 Å, from 39.0 Å to 35.8 Å (Fig. 3*B*). This decrease in the Py-Im–induced base rise is similar to the average rise of 1-bp in B-form DNA (3.4 Å) (44). Consistently, we found that the base pair per turn has been increased from 10.0 (apo Pol II EC) to 10.8 (Pol II–Py-Im encounter complex), which is about 1 bp, upon Py-Im binding (*SI Appendix*, Table S2). Taken together, these results revealed how Py-Im induces 1-bp squeezing between the active site and Rpb5 Jaw motif of Pol II.

### Binding of Py-Im Prevents Pol II Forward Translocation and Induces Pol II Backtracking.

**Fig. 4.**
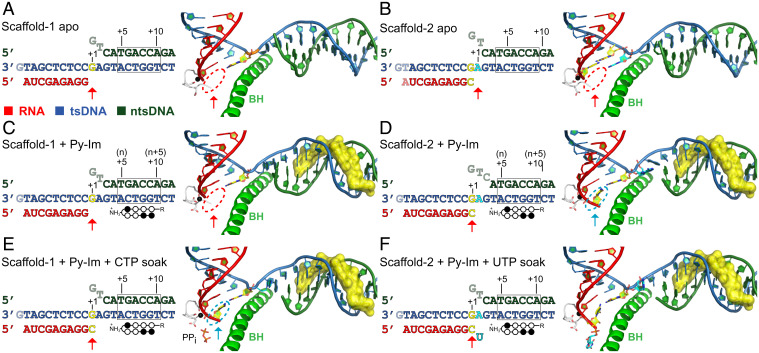
Stepwise structural snapshots of Py-Im–induced transcriptional arrest revealed by six distinct structures using scaffold-1 (*Left*) and scaffold-2 (*Right*). Red and cyan arrows indicate the +1 position of each structure. Note that for the posttranslocation state, the 3′-RNA is located at the −1 position and the active site at the +1 position is empty (highlighted in red circles); for the pretranslocation state, the 3′-RNA occupies the +1 position (highlighted in cyan circles). (*A* and *B*) Canonical Pol II elongation complexes at two consecutive posttranslocation states in the absence of Py-Im (controls): Pol II elongation complex with scaffold-1 with a 9mer RNA (scaffold-1 apo, *A*); Pol II elongation complex with scaffold-2 with a 10mer RNA (scaffold-2 apo, *B*). The empty active site is shown in red circles. (*C* and *D*) Two Pol II complexes in the presence of Py-Im were determined: Py-Im encounter complex (n-5) (*C*, posttranslocation state [with red circle]) and Py-Im engaged complex (n-4) (*D*, pretranslocation state [with cyan circle]). (*E*) Engaged state:ppi (n-4) was captured in a pretranslocation state by soaking the crystal of the Py-Im encounter complex (n-5) with CTP. The newly incorporated CMP occupies the +1 position (shown in cyan circle). (*F*) Backtracked state (n-3) was captured by soaking the crystal of the engaged complex (n-4) with UTP. Color code is the same as in Fig. 2. A total of 7-bp of downstream DNA duplex was omitted for clarity.

To understand how Py-Im affects Pol II forward translocation from the n-5 to the n-4 position, we determined the crystal structures of Pol II elongation complexes using two different scaffolds in the absence or presence of hairpin Py-Im **1**. Scaffold-1 is associated with a 9mer RNA (n-5, Fig. 4*A*), whereas scaffold-2 is associated with a 10mer RNA (n-4, Fig. 4*B*). As controls, we first solved the structures of two canonical Pol II elongation complexes (scaffold-1 and scaffold-2) in the absence of hairpin Py-Im **1**. These Pol II elongation complexes are in two consecutive posttranslocation states, mimicking Pol II 1-bp forward translocation after RNA extension from a 9mer (scaffold-1) to a 10mer (scaffold-2) (Fig. 4 *A* and *B*). In both posttranslocation states, the 3′-RNA end is translocated into the −1 position and the Pol II active site is available for nucleotide substrate binding for a new round of incorporation (red circles in Fig. 4 *A* and *B*).

We then determined the crystal structures of Pol II complexes in the presence of hairpin Py-Im **1**. Intriguingly, we found a very distinct scenario in the presence of Py-Im. As shown in Fig. 4*C*, the Pol II–Py-Im complex with scaffold-1 (i.e., encounter complex at n-5) is in a posttranslocation state. In sharp contrast to the apo Pol II EC with scaffold-2, we found that new Pol II–Py-Im EC with scaffold-2 is in the pretranslocation state in the presence of Py-Im (Fig. 4*D*). In the pretranslocation state, the 3′-end of RNA transcript still occupies the Pol II active site at the +1 position (cyan circle in Fig. 4*D*). We refer to this Py-Im–induced pretranslocation state as the “engaged complex (n-4)” (Fig. 4*D*).

To test whether we can observe a nucleotide addition reaction from the Pol II–Py-Im encounter complex (n-5) in crystal, we soaked the crystal of the encounter complex (scaffold-1, n-5) with cytidine triphosphate (CTP) overnight. The structure we captured reveals a postchemistry state. In this state, the RNA transcript product is extended from a 9mer to a 10mer by Pol II via a S_N_2 reaction. As a result, the matched cytidine monophosphate (CMP) is incorporated at the 3′-end of RNA transcript. Pol II is in a pretranslocation state in which the newly added CMP still occupies the active site at the +1 position (cyan circles in Fig. 4*E*). Interestingly, the pyrophosphate group is not yet released from the Pol II active site. We refer to this state as the n-4:ppi state (Fig. 4*E*). Taking together the results from the structural studies of both the engaged complex (n-4) and n-4:ppi states, we found that the pretranslocation state at the n-4 position is energetically favored in the presence of Py-Im (Fig. 4 *D* and *E*). The 3′-end of RNA transcript occupies the active site. Consequently, the next round of nucleotide binding and incorporation is greatly compromised. Therefore, this structure provides a structural explanation for why the extension from the n-4 state to the n-3 state is very slow.

To gain further structural insights into how Pol II extends from the n-4 state to the n-3 state (despite being slow), we soaked the crystals of the engaged complex (n-4) with matched uridine triphosphate (UTP) overnight to test whether we could force slow extension in the presence of hairpin Py-Im **1**, and if so, what the dominant state of Pol II would be. Interestingly, we found that UTP can be incorporated into the RNA strand to form 11mer in crystal by overnight soaking. However, this newly uridine monophosphate (UMP)-incorporated Pol II–Py-Im EC is adopted in a backtracked state (Fig. 4*F*). This indicates even though the Pol II engaged complex (n-4) dominates at the pretranslocation state in crystal, Pol II may slowly and transiently move forward to incorporate UTP. Once UMP is incorporated, Pol II moves backward, indicating the backtracked state is energetically favored in the presence of Py-Im. We refer to this Py-Im–induced backtracked state as the “backtracked state (n-3)” (Fig. 4*F*). This structure also explains why the n-3 state is a much stronger arrest state (in comparison with n-5 and n-4 states) and further extension to the n-2 state would be expected to be much slower.

### Pol II Gets Stuck on a Molecular Treadmill in the Presence of Py-Im and TFIIS.

**Fig. 5.**
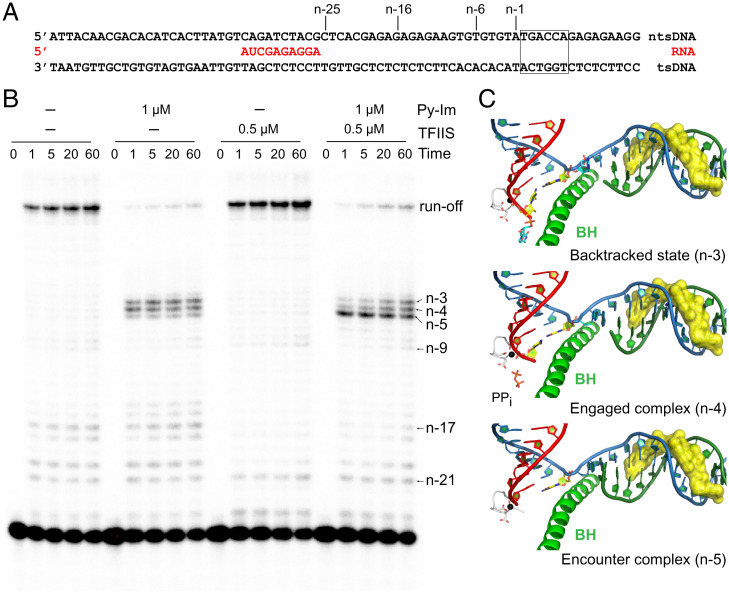
TFIIS cannot rescue Py-Im–induced strong transcriptional arrest. (*A*) The full-bubble scaffold for transcription assay. (*B*) TFIIS can stimulate RNA cleavage but fails to rescue Py-Im–induced transcriptional arrest. Presence of Py-Im induces strong arrest at n-5 to n-3 positions. Addition of TFIIS to the system significantly increases the band at the n-5 position. This indicates the majority of the n-3 complex is in a backtracked state, therefore allowing a 2-nt cleavage. (*C*) The corresponding Py-Im–arrested Pol II structures (n-5, n-4, and n-3) are shown. Color code is the same as in Fig. 2.

**Fig. 6.**
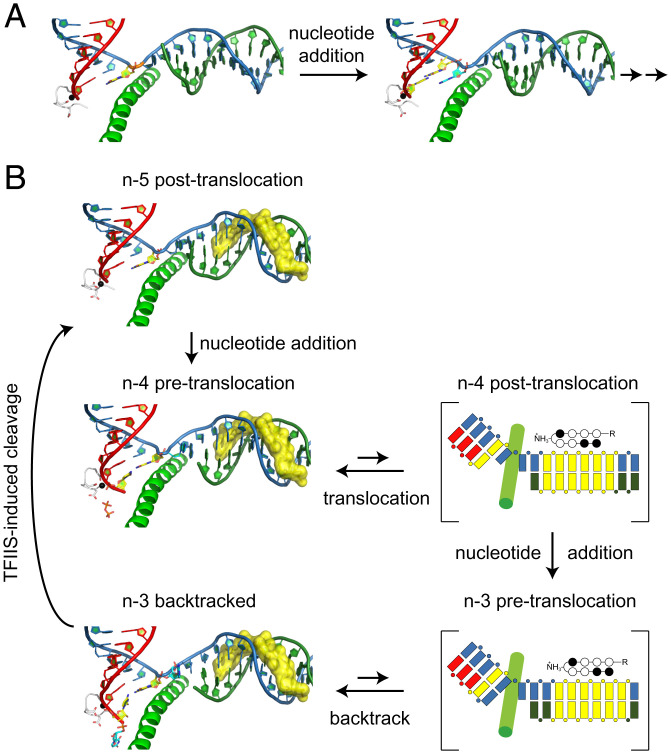
Molecular mechanism of Py-Im–induced transcriptional arrest. (*A*) Pol II incorporates incoming nucleotide and moves forward effectively in the absence of Py-Im. (*B*) Pol II is trapped among three states in the presence of Py-Im. Pol II undergoes futile cycles of nucleotide addition and cleavage in the presence of Py-Im and TFIIS.

TFIIS promotes RNA transcript cleavage at the backtracked state or pretranslocation state to generate a new posttranslocation state (45, 46). Therefore, based on the structures of the engaged complex (n-4, pretranslocation state) and backtracked state (n-3), we can have a clear predication that the RNA transcripts in these states are readily cleaved 1 nt and 2 nt by TFIIS, respectively, to generate a new posttranslocation state at n-5. To test this, we performed a transcription assay in the presence or absence of hairpin Py-Im **1** and TFIIS. Consistent with our previous report, TFIIS cannot rescue Py-Im–induced transcriptional arrest even with prolonged incubation (up to 2 h, Fig. 5) (29). However, we noticed the pattern of pausing/arrest bands undergoes a significant change in the presence of TFIIS. While the major pausing bands correspond to n-3 and n-4, followed by the n-5 band, in the absence of TFIIS, the major pausing band shifts toward the n-5 band in the presence of TFIIS (Fig. 5). This result fully supports our prediction that the n-4 state (engaged complex) and the n-3 state (backtracked state) are more prone to TFIIS cleavage, whereas the n-5 state (encounter state at the posttranslocation state) is resistant to TFIIS cleavage. Taking together the results from our biochemical and structural studies, we are now able to attribute the three consecutive stalling bands to the three defined Py-Im–induced Pol II–arrested structures (Fig. 5). We also obtained structural insights into why TFIIS fails to rescue Py-Im–induced Pol II arrest. Py-Im represents a class of very stable and bulky barriers. The binding affinity of Py-Im to its target DNA is extremely high (with nanomolar scale) (47, 48). The off-rate of Py-Im from Pol II elongation complex is extremely slow (even slower than the dsDNA). Indeed, we found that Pol II cannot bypass the Py-Im barriers even after over 40 h of incubation time (29). The presence of TFIIS leads to futile cycles of slow nucleotide addition and TFIIS-stimulated RNA transcript cleavage without bypassing the Py-Im barriers. Pol II may slowly extend from the n-5 state to the n-4 or n-3 state, both of which (either in pretranslocation or backtracked state) are prone to TFIIS cleavage, and return to the n-5 state, which is resistant to further cleavage. As a result, Pol II is trapped by the Py-Im barrier, such that it is stuck on a “molecular treadmill” (Fig. 6).

In summary, we investigated the molecular mechanism of transcriptional arrest by a noncovalent DNA-binding small molecule, pyrrole-imidazole polyamide **1**. Our structural studies captured stepwise structural snapshots at distinct states of Py-Im **1**–induced Pol II–arrested complexes, including one n-5 state (encounter complex, posttranslocation state), two n-4 states that are trapped at the pretranslocation state (engaged complex, n-4:ppi), and one n-3 state (Py-Im–induced backtracked state). Our structural and functional analyses provide important structural insights into how Py-Im **1** is bound within the Pol II elongation complex and interacts with Pol II; how Py-Im **1** leads to conformational changes of downstream DNA duplex; how bound Py-Im **1** prevents Pol II forward translocation and induces backtracking; how Py-Im **1** traps Pol II into persistent arrest; and why TFIIS fails to rescue Py-Im–induced arrest. Our study may also have implications in targeting transcriptional addiction for cancer therapy with small molecules (49–51).

## Materials and Methods

Detailed descriptions of synthesis of hairpin Py-Im **1** and purification of RNA Pol II are provided in *SI Appendix*. For in vitro transcription assay with a miniscaffold, 200 nM of 5′-^32^P-labeled RNA (5′-AUCGAGAGG-3′), 600 nM of TS DNA (5′-CCTTCTCTCTGGTCATGAGCCTCTCGATG-3′), and 800 nM of nontemplate strand DNA (5′-GTCATGACCAGAGAGAAGG-3′) was annealed in elongation buffer to prepare the miniscaffold. Py-Im was dissolved in dimethylsulfoxide, and the concentration was validated by measuring the absorbance at 310 nm using a NanoDrop. Various concentrations of Py-Im were added to the miniscaffold and incubated for 3 h at room temperature. The prepared miniscaffold was then mixed with Pol II and preincubated for 10 min at room temperature to assemble the EC. Reaction was started by mixing equal volume of EC and ribonucleoside triphosphate (rNTP) or TFIIS and was quenched by adding quench-loading buffer (90% formamide, 50 mM ethylenediaminetetraacetic acid (EDTA), 0.05% xylene cyanol, and 0.05% bromophenol blue) and analyzed by 12% urea/tris/borate/EDTA polyacrylamide gel electrophoresis (TBE PAGE). For transcription assays with a full-bubble scaffold, the scaffold was assembled by annealing TS DNA and RNA, followed by the addition of Pol II and incubated for 20 min at room temperature. After adding nontemplate strand DNA and incubating for 10 min, transcription was initiated as described above. All transcription assays were repeated three times. For structure determination of Py-Im–bound dsDNA complexes, an aliquot of 0.7 mM duplex DNA:Py-Im was mixed with an equal volume of crystallization solution containing 10 mM Tris (pH 7.5), 24% 2-methyl-2,4-pentanediol (MPD), and 35 mM calcium acetate, with 35% MPD as a reservoir. Crystals were obtained by the sitting-drop vapor diffusion method at 4 °C for 4 to 6 wk. For structural determination of Py-Im–bound Pol II elongation complex, the crystals of Py-Im–bound Pol II elongation complexes were obtained by hanging-drop methods with incubation, with crystallization buffer (390 mM ammonium phosphate [pH 6.0], 5 mM dithiothreitol [DTT], 5 mM dioxane, and 9 to 13% [wt/vol] polyethylene glycol [PEG 6,000]) at 22 °C for 7 to 14 d. A detailed description of structural determination and refinement can be found in *SI Appendix*.

## Supplementary Material

Supplementary File

## Data Availability

All atomic coordinates and structure factors are deposited at the Research Collaboratory for Structural Bioinformatics Protein Data Bank (PDB). All PDB codes are listed in *SI Appendix*, Table S1. Structure coordinates data have been deposited in the PDB (7RIL, 7RIQ, 7RIM, 7RIP, 7RIW, 7RIX, and 7RIY). All other study data are included in the article and/or *SI Appendix*.
