# Supplementary material for: RNA polymerase II trapped on a molecular treadmill: Structural basis of persistent transcriptional arrest by a minor groove DNA binder
Source: Proc Natl Acad Sci U S A. 2022 Jan 12;119(3):e2114065119. doi: 10.1073/pnas.2114065119 (PMC8784135; doi:10.1073/pnas.2114065119)
Supplement: Supplementary File [file pnas.2114065119.sapp.pdf]

Supplementary Information for

RNA polymerase II trapped on a molecular treadmill: structural basis of persistent transcriptional arrest by a minor groove DNA binder

Juntaek Oh[1], Tiezheng Jia[2], Jun Xu[1], Jenny Chong[1], Peter B. Dervan[2,*] and Dong Wang[1,3,4,*]

[1]Division of Pharmaceutical Sciences, Skaggs School of Pharmacy & Pharmaceutical Sciences; University of California, San Diego, La Jolla, California 92093, United States

[2] Division of Chemistry and Chemical Engineering, California Institute of Technology, Pasadena, California 91125, United States

[3]Department of Cellular and Molecular Medicine, University of California, San Diego, La Jolla, California 92093, United States

[4]Department of Chemistry and Biochemistry, University of California, San Diego, La Jolla, California 92093, United States

[*]To whom correspondence should be addressed. Email: dongwang@ucsd.edu (DW); dervan@caltech.edu (PBD). DW: https://orcid.org/0000-0002-2829-1546

This PDF file includes:

- Materials and Methods
- Figures S1-S2
- Table S1-S2
- References for SI

Materials and Methods

Synthesis and purification of hairpin Py-Im 1

Hairpin Py-Im **1** was synthesized on solid support as previously described (1-3). All compounds were purified by RP-HPLC and correct masses were verified by MALDI-TOF.

In vitro transcription assay

Ten-subunit RNA Pol II was purified as previously described (4-6). For transcription assay, 12-subunit Pol II was prepared by adding four molar excesses of Rpb4 and Rpb7, followed by size exclusion chromatography. Final purification buffer was 20 mM Tris (pH 7.5), 40 mM KCl, 5 mM $MgCl_2$, and 5 mM DTT (elongation buffer). Transcription assay was performed as previously described (7, 8). Briefly, reconstituted 12-subunit Pol II was used for transcription assays. 200 nM of 5′-^{32}P-labeled RNA (5′-AUCGAGAGG-3′), 600 nM of template strand DNA (5′-CCTTCTCTCTGGTCATGAGCCTCTCGATG-3′) and 800 nM of non-template strand DNA (5′-GTCATGACCAGAGAGAAGG-3′) was annealed in elongation buffer to prepare the mini-scaffold. Py-Im was dissolved in DMSO, and the concentration was validated by measuring the absorbance at 310 nm using a NanoDrop. Various concentration of Py-Im was added to the mini-scaffold and incubated for 3 hours at room temperature. Prepared mini-scaffold was mixed with Pol II and preincubated for 10 min at room temperature to assemble the elongation complex (EC). Reaction was started by mixing equal volume of EC and rNTP or TFIIS. Final concentrations were 20 nM of mini-scaffold, 120 nM of Pol II, 0-1 mM of rNTP, and 0-1 μM of TFIIS. Reaction was quenched by adding quench-loading buffer (90% formamide, 50 mM EDTA, 0.05% xylene cyanol, and 0.05% bromophenol blue) and analyzed by 12% urea/TBE PAGE.

For transcription assays with a full-bubble scaffold, the scaffold was assembled by annealing tsDNA and RNA, followed by the addition of Pol II and incubated for 20 min at room temperature. After adding ntsDNA and incubating for 10 min, transcription was initiated as described above. All transcription assays were repeated three times.

Crystallization of Py-Im bound dsDNA complex

HPLC-purified DNA oligonucleotides for crystallization was purchased from IDT. Sequence of template strand DNA was 5′-CCTGGTCAGG-3′ and non-template strand DNA was 5′-CCTGACCAGG-3′. Crystallization method was similar with previous reports, with minor modification (9, 10). Duplex DNA was dissolved in 10 mM Tris (pH 7.5) and annealed by heating at 65 °C for 5 min and cooling down to room temperature. Equivalent molar ratio of Py-Im was added to duplex DNA and incubated for 3 hours at room temperature. Prior to crystal setup, duplex DNA and Py-Im mixture was centrifuged at 13,000 RPM for 10 min. An aliquot of 0.7 mM duplex DNA:Py-Im was mixed with an equal volume of crystallization solution containing 10 mM Tris (pH 7.5), 24% 2-methyl-2,4-pentanediol (MPD) and 35 mM calcium acetate, with 35% MPD as a reservoir. Crystals were obtained by sitting-drop vapor diffusion method at 4 °C for 4-6 weeks. Crystals were moved to cryo-solution (10 mM Tris (pH 7.5), 35% MPD and 35 mM calcium acetate) and incubated for at least 30 min prior to flash-freezing in liquid nitrogen.

Crystallization of Py-Im bound Pol II elongation complex

Ten-subunit Pol II was crystallized as previously described (8). Mini-scaffold was prepared by annealing 1:2:2 molar ratio of tsDNA, RNA, and ntsDNA in elongation buffer. Four molar ratios of Py-Im was added and incubated for 3 hours at room temperature. Elongation complex was

prepared by incubating 3 μM of Pol II and 12 μM of the mini-scaffold at room temperature for 30 min and 4 °C for 30 min. To change buffer and remove excess amount of DNA/RNA, three rounds of ultrafiltration were performed by adding equal volume of final buffer (25 mM Tris (pH 7.5), 20 mM NaCl, 5 mM DTT, 1 μM $Zn(OAc)_2$, 100 μM EDTA, 10 μM Py-Im). EC was concentrated to 6-8 mg/mL. Hanging drop with crystallization buffer (390 mM ammonium phosphate (pH 6.0), 5 mM DTT, 5 mM dioxane, and 9-13 % (w/v) PEG 6,000) produced crystals for diffraction after 7-14 days of incubation at 22 °C. Crystals were transferred to cryo solution (100 mM MES (pH 6.0), 350 mM NaCl, 5mM DTT, 5mM Dioxane, 16 % PEG 6,000, and 17 % PEG400) and incubated at 4 °C for overnight and flash-frozen in liquid nitrogen (6). For CTP and UTP soaking, 10 mM of CTP or UTP together with 10 mM $MgCl_2$ was added to cryo solution and incubated at 4 °C for overnight.

Structure determination and refinement

X-ray diffraction datasets were collected at beamlines 5.0.1, 8.2.1 and 8.2.2, Advanced Light Source, Lawrence Berkeley National Laboratory. For Py-Im dsDNA complex structure determination, collected images were processed by iMosflm (11). CC1/2 higher than 0.3 was used for high resolution determination (12). Space group of Py-Im dsDNA crystals was P3, with one complex in the asymmetric unit. We performed molecular replacement using Phenix with "ideal" double stranded B-form DNA prepared by Coot as an initial search model (13, 14). Several rounds of manual model building and refinement were performed by using Phenix and Coot.

For Py-Im Pol II complex structure determination, images were processed by XDS, followed by pointless and aimless (15, 16). All crystals had space group C2, with one elongation complex in

the asymmetric unit. Molecular replacement was performed for phasing using Pol II EC as a search model (PDB ID: 6UQ2) (8). Initial refinement was performed without downstream DNA, to get unbiased electron density. For Py-Im model building, downstream DNA duplex was modeled first, aligned the Py-Im–dsDNA complex to downstream, and subsequently used high resolution Py-Im structure as a starting model for Pol II-EC-Py-Im building. Several rounds of manual building and refinement were performed using Phenix and Coot to get the final structure with the best fit to the electron density (13, 14). Data collection and refinement statistics are summarized in Table S1. Figures with structural information are prepared by using Pymol (17).

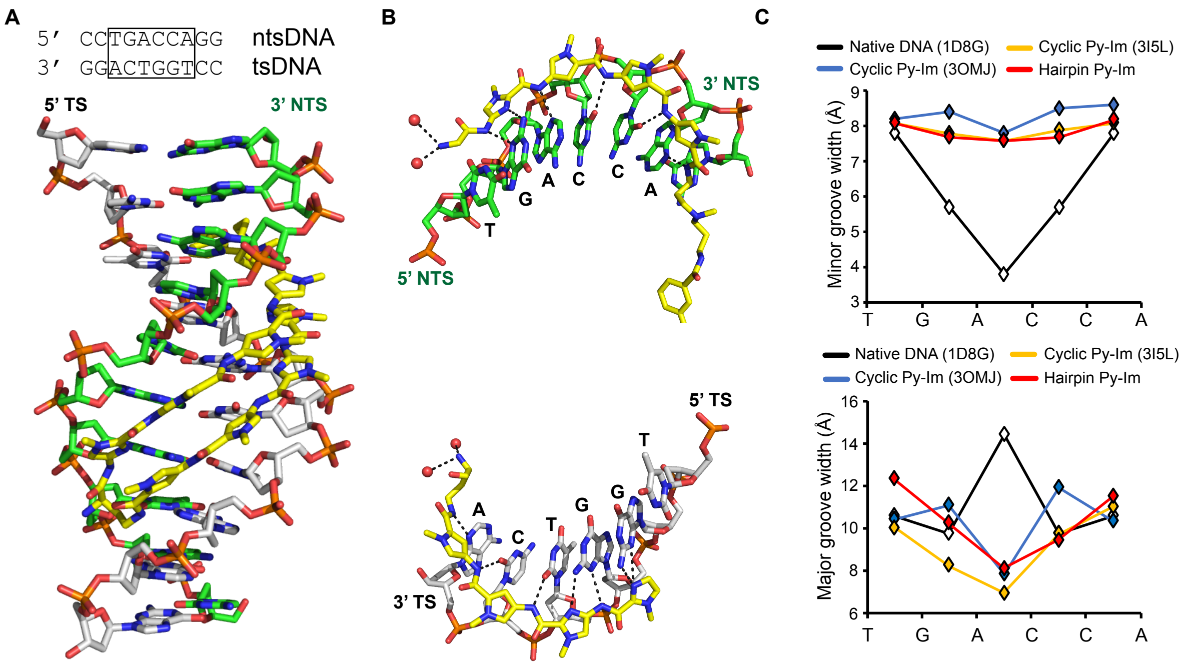

Figure S1. Crystal structure of Py-Im-dsDNA complex. (A) Overall structure of Py-Im-dsDNA complex. (B) Hydrogen bonding network between NTS strand and half of Py-Im (top panel) and that of between TS strand and another half of Py-Im (bottom panel). (C) Minor groove and major groove width analysis. Hairpin Py-Im **1** (this study), native DNA (PDB id: 1D8G) and two different cyclic Py-Im complexed with dsDNA (PDB id: 3I5L, 3OMJ) are shown.

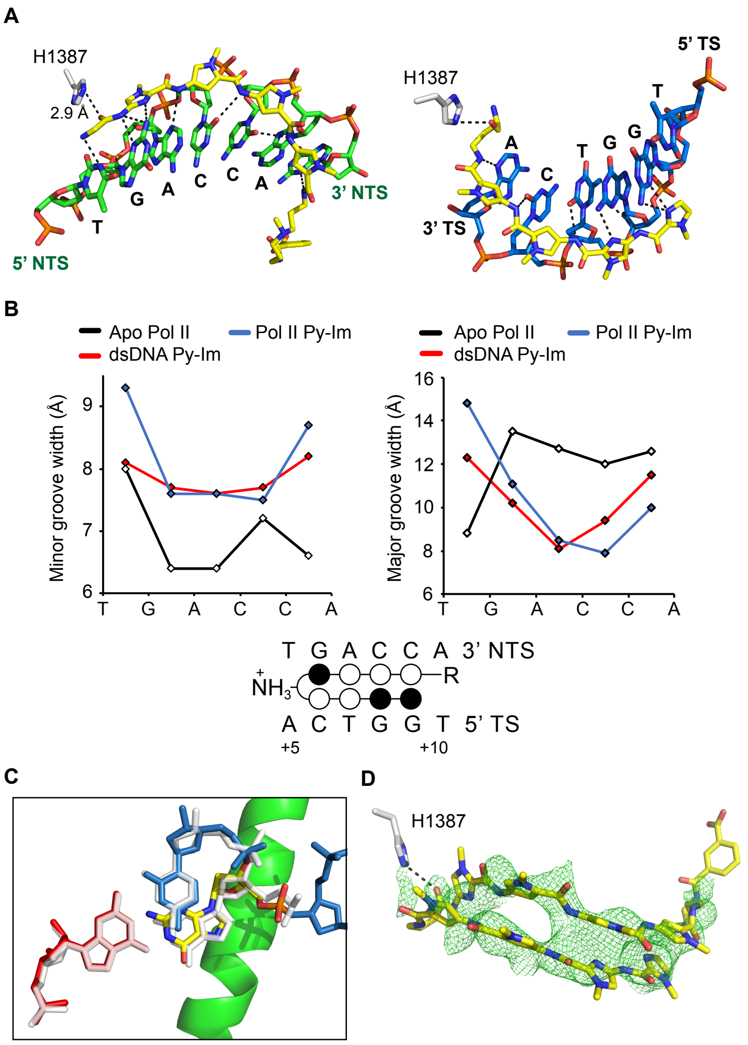

Figure S2. Analysis of Py-Im Pol II interaction. (A) Hydrogen bonding network between NTS strand and half of Py-Im (left panel) and that of between TS strand and another half of Py-Im (right panel). (B) Minor and major groove width analysis of apo Pol II, Pol II Py-Im and Py-Im-dsDNA structures. Substantial minor groove widening and major groove contraction was observed. (C) Structure superposition of apo Pol II and Encounter complex. Apo Pol II is shown in white. (D) Composite omit Fo-Fc electron density map of Py-Im in Encounter complex is contoured at 2.5 σ. Phenix composite omit map was used to prepare electron density (14).

Table S1. Data collection and refinement statistics

	Py-Im dsDNA	Pol II EC apo (Sca-1)	Pol II-Py-Im (Sca-1)	Pol II-Py-Im CTP soak (Sca-1)	Pol II EC apo (Sca-2)	Pol II-Py-Im (Sca-2)	Pol II-Py-Im CTP soak (Sca-2)
PDB ID	7RIL	7RIQ	7RIM	7RIP	7RIW	7RIX	7RIY
**Data collection**							
Space group	P 31	C 1 2 1					
a, *b*, *c* (Å)	33.9 33.9 47.5	168 223 193	167 222 194	168 223 193	165 222 192	168 222 194	168 223 193
α, β, γ (°)	90 90 120	90 100 90	90 100 90	90 101 90	90 100 90	90 100 90	90 101 90
Resolution (Å)*	29.4 - 1.8 (1.86 - 1.8)	48.2 - 3.0 (3.11 - 3.0)	82.4 - 2.9 (3.00 - 2.9)	48.2 - 3.3 (3.42 - 3.3)	46.6 - 3.2 (3.31 - 3.2)	49.4 - 3.4 (3.52 - 3.4)	49.55 - 3.7 (3.83 - 3.7)
Unique reflections	5661 (555)	139507 (13966)	153630 (15294)	105034 (10490)	112410 (11266)	96080 (9563)	74192 (7420)
Multiplicity	33.6 (32.6)	2.0 (2.0)	2.0 (2.0)	2.0 (2.0)	2.0 (2.0)	2.0 (2.0)	2.0 (2.0)
Completeness (%)	99.8 (99.8)	99.8 (99.3)	99.7 (98.1)	99.8 (99.3)	99.8 (99.2)	99.8 (99.5)	99.8 (99.8)
Mean I/sigma(I)	104.3 (1.8)	5.6 (0.6)	5.8 (0.6)	6.9 (0.7)	5.5 (0.7)	6.0 (1.0)	4.7 (0.9)
R-merge	0.702 (2.604)	0.089 (1.204)	0.117 (1.437)	0.137 (1.215)	0.140 (1.327)	0.155 (1.03)	0.160 (0.994)
CC1/2	0.874 (0.717)	0.995 (0.415)	0.99 (0.299)	0.989 (0.301)	0.987 (0.276)	0.982 (0.454)	0.981 (0.321)
**Refinement**							
No. reflections	5648 (554)	139260 (13870)	153148 (14997)	104906 (10417)	112221 (11174)	95948 (9520)	74096 (7403)
R_{work} / R_{free}	0.204 / 0.229	0.213 / 0.264	0.216 / 0.251	0.219 / 0.260	0.228 / 0.265	0.222 / 0.268	0.232 / 0.281
No. atoms							
Macromolecules	404	29246	29083	29076	29069	29052	29060
Ligands	103	9	108	117	9	108	108
RMS (bonds, Å)	0.016	0.004	0.007	0.006	0.005	0.007	0.006
RMS (angles, °)	1.96	0.95	1.23	1.18	0.99	1.27	1.05
Clashscore	6.68	10.20	20.33	13.13	12.50	13.12	17.14
Average B-factor	63.35	110.22	99.40	111.46	100.28	98.87	119.40
Macromolecules	60.82	110.20	99.12	111.04	100.27	98.42	119.00
Ligands	73.50	183.56	174.92	214.74	128.85	220.83	227.66

*Values in parentheses are for the highest-resolution shell.

Table S2. The base twist parameters of DNA duplexes observed in Pol II, Pol II-Py-Im, and Py-Im-dsDNA crystals

	Pol II		dsDNA
nts step	apo	Py-Im	Py-Im
CA	34.1	25.4	31.7
AT	28.2	41.3	33.8
TG	41.8	23.8	30.9
GA	36.8	38.6	39.3
AC	34.7	32.9	33.8
CC	32.4	35.4	36.3
CA	36.3	42.2	37.4
AG	35.2	37.9	26.0
GA	38.0	21.9	36.4
AG	37.9	33.3	
GA	39.6	33.9	
AG	32.6	33.9	
GA	39.2	32.9	
Mean ± SD	35.9 ± 3.6	33.3 ± 6.3	34.0 ± 4.0
Base per turn	10.0	10.8	10.6

SI References

1. C. Dose, M. E. Farkas, D. M. Chenoweth, P. B. Dervan, Next generation hairpin polyamides with (R)-3,4-diaminobutyric acid turn unit. *J Am Chem Soc* **130**, 6859-6866 (2008).
2. J. W. Puckett, J. T. Green, P. B. Dervan, Microwave assisted synthesis of Py-Im polyamides. *Org Lett* **14**, 2774-2777 (2012).
3. B. C. Li, D. C. Montgomery, J. W. Puckett, P. B. Dervan, Synthesis of cyclic Py-Im polyamide libraries. *J Org Chem* **78**, 124-133 (2013).
4. D. Wang, D. A. Bushnell, K. D. Westover, C. D. Kaplan, R. D. Kornberg, Structural basis of transcription: role of the trigger loop in substrate specificity and catalysis. *Cell* **127**, 941-954 (2006).
5. D. Wang *et al.*, Structural basis of transcription: backtracked RNA polymerase II at 3.4 angstrom resolution. *Science* **324**, 1203-1206 (2009).
6. J. Oh, J. Xu, J. Chong, D. Wang, Structural and biochemical analysis of DNA lesion-induced RNA polymerase II arrest. *Methods* 10.1016/j.ymeth.2019.02.019 (2019).
7. W. Wang, C. Walmacq, J. Chong, M. Kashlev, D. Wang, Structural basis of transcriptional stalling and bypass of abasic DNA lesion by RNA polymerase II. *Proc Natl Acad Sci U S A* **115**, E2538-E2545 (2018).
8. J. Oh *et al.*, RNA polymerase II stalls on oxidative DNA damage via a torsion-latch mechanism involving lone pair-pi and CH-pi interactions. *Proc Natl Acad Sci U S A* **117**, 9338-9348 (2020).
9. D. M. Chenoweth, P. B. Dervan, Allosteric modulation of DNA by small molecules. *Proc Natl Acad Sci U S A* **106**, 13175-13179 (2009).
10. D. M. Chenoweth, P. B. Dervan, Structural basis for cyclic Py-Im polyamide allosteric inhibition of nuclear receptor binding. *J Am Chem Soc* **132**, 14521-14529 (2010).
11. T. G. Battye, L. Kontogiannis, O. Johnson, H. R. Powell, A. G. Leslie, iMOSFLM: a new graphical interface for diffraction-image processing with MOSFLM. *Acta Crystallogr D Biol Crystallogr* **67**, 271-281 (2011).
12. P. A. Karplus, K. Diederichs, Linking crystallographic model and data quality. *Science* **336**, 1030-1033 (2012).
13. P. Emsley, K. Cowtan, Coot: model-building tools for molecular graphics. *Acta Crystallogr D Biol Crystallogr* **60**, 2126-2132 (2004).
14. P. D. Adams *et al.*, PHENIX: a comprehensive Python-based system for macromolecular structure solution. *Acta Crystallogr D Biol Crystallogr* **66**, 213-221 (2010).
15. W. Kabsch, Xds. *Acta Crystallogr D Biol Crystallogr* **66**, 125-132 (2010).
16. P. R. Evans, G. N. Murshudov, How good are my data and what is the resolution? *Acta Crystallogr D Biol Crystallogr* **69**, 1204-1214 (2013).
17. Schrodinger, LLC (2015) The PyMOL Molecular Graphics System, Version 1.8.
